# Lung abscess following ventilator-associated pneumonia during COVID-19: a retrospective multicenter cohort study

**DOI:** 10.1186/s13054-023-04660-x

**Published:** 2023-10-04

**Authors:** S. Hraiech, K. Ladjal, C. Guervilly, H. Hyvernat, L. Papazian, J. M. Forel, A. Lopez, N. Peres, J. Dellamonica, M. Leone, I. Gragueb-Chatti

**Affiliations:** 1 Service de Médecine Intensive - Réanimation, AP-HM, Hôpital Nord, Marseille, France; 2 Faculté de Médecine, Aix-Marseille Université, Centre d’Etudes Et de Recherches Sur Les Services de Santé Et Qualité de Vie EA 3279, 13005 Marseille, France; 3 CHU de Nice, Hôpital Archet 1, Médecine Intensive Réanimation, 06200 Nice, France; 4 Equipe 2 CARRES, UR2CA, Unité de Recherche Clinique Côte d’Azur, Université Côte d’Azur, Nice, France; 5 Centre Hospitalier de Bastia, 20600 Bastia, Corsica France; 6 Service d’Anesthésie Et de Réanimation, Aix Marseille Université, Assistance Publique Hôpitaux Universitaires de Marseille, Hôpital Nord, Marseille, France; 7 Service de Réanimation Polyvalente, Centre Hospitalier Intercommunal Toulon – La Seyne sur Mer, Toulon, France

**Keywords:** COVID-19, Ventilator-associated pneumonia, Lung abscess

## Abstract

**Background:**

Patients undergoing mechanical ventilation (MV) for COVID-19 exhibit an increased risk of ventilator-associated pneumonia (VAP). The occurrence of lung abscesses following VAP in these patients has been poorly studied. We aimed to describe the incidence, characteristics, risk factors and prognosis of lung abscesses complicating VAP after COVID-19.

**Methods:**

We conducted an observational, retrospective study in three French intensive care units. Patients admitted for acute respiratory failure with a confirmed SARS-CoV-2 PCR and requiring MV for more than 48 h were included.

**Results:**

Among the 507 patients included, 326 (64%) had a documented VAP. Of these, 23 (7%) developed a lung abscess. Enterobacterales (15/23, 65%) were the main documentation, followed by non-fermenting Gram-negative bacilli (10/23, 43%) and Gram-positive cocci (8/23, 35%). Lung abscesses were mainly plurimicrobial (15/23, 65%). In multivariate analysis, a plurimicrobial 1st VAP episode (OR (95% CI) 2.93 (1.16–7.51); p = 0.02) and the use of hydrocortisone (OR (95% CI) 4.86 (1.95–12.1); p = 0.001) were associated with lung abscess development. Intensive care unit (ICU) mortality of patients with lung abscesses reached 52%, but was not significantly higher than for patients with VAP but no lung abscess. Patients with lung abscesses had reduced ventilator-free days at day 60, a longer duration of MV and ICU stay than patients with VAP but no lung abscess (respectively, 0 (0–3) vs. 16 (0–42) days; p < 0.001, 49 (32–73) vs. 25 (11–41) days; p < 0.001, 52 (36–77) vs. 28 (16–47) days; p < 0.001).

**Conclusions:**

Lung abscessing pneumonia is not uncommon among COVID-19 patients developing VAP. A plurimicrobial first VAP episode and the use of hydrocortisone are independently associated with this complication. In COVID-19 patients with persistent VAP, a chest CT scan investigating the evolution toward lung abscess should be considered.

**Supplementary Information:**

The online version contains supplementary material available at 10.1186/s13054-023-04660-x.

## Background

According to WHO last updates, there has been more than 6 M deaths from Corona Virus Disease 19 (COVID-19) worldwide since the beginning of pandemic [1]. Mortality is mainly due to the severity of pulmonary injury. SARS-CoV-2 pneumonia may progress toward acute respiratory distress syndrome (ARDS) requiring intensive care unit (ICU) admission and frequent recourse to mechanical ventilation (MV) [2].

Among patients under invasive MV, COVID-19 is associated with an increased risk of ventilator-associated pneumonia (VAP). Rouze et al. describe an incidence of 50.5% in a large European cohort [3], this incidence raising 86%, in patients under ECMO [4].

Prolonged MV, virus-induced lung vascular injury [5] and host defense decrease [6] as well as the large use of immunosuppressive therapies have been mentioned to explain increased risk of VAP during COVID-19 [7].

Lung abscess is a rare but serious complication of community-acquired or nosocomial pneumonia. Its incidence among COVID-19 patients experiencing VAP is uncertain as data specifically focusing on this issue are mainly cases reports [8]. Series describing VAP after COVID-19 report infrequent occurrence of lung abscess with an incidence as low as 1.4% [9]. Only 2 single-center studies have specifically assessed the question [10, 11] and reported a 14 to 20% incidence of lung abscesses after VAP. However, risk factors and outcomes of such a complication remain poorly investigated.

We therefore conducted a retrospective multicenter trial to describe the incidence, clinical and microbiological characteristics, risk factors and outcomes of patients developing lung abscess under MV following COVID-19 ARDS.

## Methods

### Study design and population

This is a retrospective, multicenter observational study carried out in 3 ICUs from French University Hospitals.

Patients admitted from March 2020 to December 2021 were included if they met the following criteria:

- Age > 18
- ARDS secondary to SARS-CoV-2 pneumonia (confirmed by nasopharyngeal or lower respiratory sample RT-PCR)
- Invasive MV for 48 h or more

### Data collection

The following data were collected from the patients’ electronic medical file:

- Demographic characteristics and severity score at ICU admission [12, 13]
- Duration of MV, ICU and hospital length of stay, mortality at D28, D90, ICU and hospital discharge
- Bacterial coinfection and antibiotic use at ICU admission
- Immunomodulatory (tocilizumab) and/or immunosuppressive treatments during the ICU stay (corticosteroids, interleukin-1 receptor antagonist (anakinra) or selective Janus kinase enzyme inhibitor (ruxolitinib)).
- VAP (up to the third episode) documentation
- Antibiotics regimen
- Chest CT scan and diagnosis of lung abscess

If a lung abscess was diagnosed, its characteristics were collected:

- Radiological characteristics: number, location, dimension, contact with the visceral pleura, visibility on the chest X-ray, pulmonary embolism or thrombosis, radiological evolution during the ICU stay.
- Microbiological data: bacterial identification, antibiotic susceptibility, associated bacteremia, concomitant pulmonary aspergillosis [14, 15]
- Antibiotics regimen
- Surgical treatment
- Associated complications (pneumothorax, empyema, hemoptysis)

### Definitions

#### VAP

VAP was diagnosed in patients having received MV for at least 48 h when the following criteria were met [16, 17]:

- New or progressive persistent infiltration on chest radiograph.
- At least two of the following: new onset of fever, purulent endotracheal aspirate, leukocytosis or leucopenia, increased minute ventilation, arterial oxygenation decline, need for increased vasopressor infusion to maintain blood pressure (for patients with ARDS, for whom demonstration of radiological deterioration is difficult, at least two of the preceding criteria sufficed).
- A positive quantitative culture from bronchoalveolar lavage (BAL), protected distal sample (PDS) or endotracheal aspirate (ETA).

A positive bacterial culture on a respiratory sample without clinical sign of pneumonia and without antibiotic treatment initiated was considered as a colonization.

#### Lung abscesses

Lung abscesses were diagnosed on CT scan based on the radiologist description.

### Statistical analysis

Analyses were performed first in the whole cohort, and then, patients were divided into 3 groups according to whether they developed a VAP and a lung abscess. The 3 groups were named:

o no VAP
o VAP without lung abscess
o VAP with lung abscess

Categorical variables were expressed as numbers and percentages. Quantitative variables were expressed as median and interquartile range (IQR) or mean and standard deviation (SD). Comparisons between patients without VAP, patients with VAP but no abscess, and patients with VAP and lung abscess groups were made by the Mann–Whitney U test for categorical variables and by the t-test for continuous variables. In case of a significant difference with comparison of more than 2 groups, post hoc tests were performed. We used the Kruskal–Wallis test for post hoc comparisons for categorical variables and the Bonferroni test for continuous variables.

Subsequently, we performed a multivariate analysis by logistic regression to identify factors associated with the occurrence of at least one VAP with abscess, including non-collinear variables whose significance threshold was p < 0.1 in univariate analysis. The number of variables included in the multivariate analysis was consistent with the number of cases observed (VAP with lung abscess). Collinearity between variables included in the logistic regression analysis was excluded with Chi-square test for categorical variables or with Pearson correlation tests for continuous variables. We used a stepwise approach for the logistic regression analysis. We did not use multiple imputation for missing data.

We performed a Hosmer–Lemeshow test to ensure the validity of the logistic regression.

The results of the multivariate analysis are expressed as odds ratios and 95% confidence intervals. The significance level was 0.05. All statistical tests were performed with SPSS 20.0 software.

## Results

### Study flowchart

**Fig. 1:**
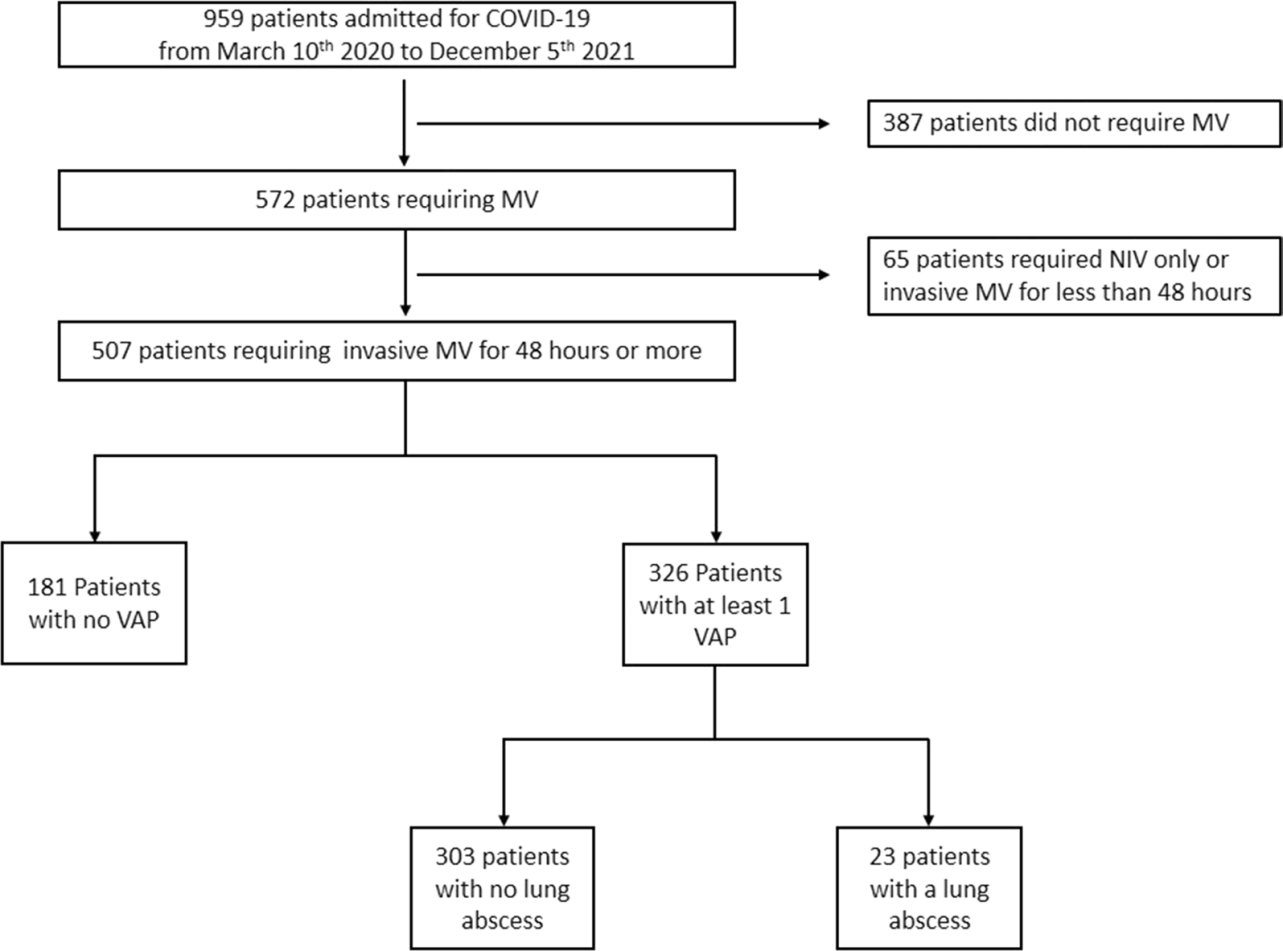
Study flowchart. *NIV* noninvasive ventilation. *MV* mechanical ventilation. *VAP* ventilator-associated pneumonia

Figure 1 presents the study flowchart. Among the 507 patients included in the final analysis, 326 had at least one episode of VAP resulting in a VAP incidence of 64%. Twenty-three patients (7%) from the VAP group developed a lung abscess. There were no missing data concerning VAP or lung abscess occurrence.

### Patients’ characteristics

Table 1 describes the patients’ characteristics in the whole cohort and subgroups at the time of ICU admission. Additional file 1: Table S1 compares the use of immunosuppressive or immunomodulatory treatments during the ICU stay between all groups.

**Table 1:** Patients’ characteristics at ICU admission

	All (n = 507)	No VAP (n = 181)	VAP without lung abscess (n = 303)	VAP with lung abscess (n = 23)	*P* value
Male, n (%)	368 (73)	125 (69)	224 (74)	19 (83)	0.277
Age, years ( mean ± SD)	62 ± 12	62 ± 12	62 ± 12	55 ± 14	**0.028**
SAPS 2 (median, IQR)	40 (32–48)	40 (31–50)	41 (34–49)	42 (34–53)	0.519
SOFA (median, IQR)	5 (3–8)	5 (3–8)	5 (3–8)	8 (4–9)	0.071
Hypertension, n (%)	243 (48)	88 (48)	143 (47)	12 (52)	0.875
Diabetes mellitus, n (%)	162 (32)	55 (30)	99 (33)	8 (35)	0.835
Obesity, n (%)	201 (40)	74 (41)	118 (39)	9 (39)	0.914
Smoker, n (%)	122 (24)	51 (28)	70 (23)	1 (4)	**0.035**
Chronic heart disease, n (%)	92 (18)	35 (19)	55 (18)	2 (9)	0.456
Chronic respiratory insufficiency, n (%)	70 (14)	21 (11)	47 (16)	2 (9)	0.371
Chronic renal failure, n (%)	42 (8)	14 (8)	27 (9)	1 (4)	0.706
Cancer, n (%)	50 (10)	16 (9)	31 (10)	3 (13)	0.771
Immunocompromised status, n (%)	60 (12)	18 (10)	39 (13)	3 (13)	0.618
Time from hospital to ICU admission, days (median, IQR)	1 (0–3)	1 (0–4)	1 (0–2)	2 (0–4)	0.160
Probabilistic antibiotic therapy, n (%)	319 (63)	129 (71)	173 (57)	17 (74)	**0.004**
Confirmed bacterial coinfection on admission, n (%)	56 (11)	13 (7)	38 (13)	5 (22)	**0.047**
Dexamethasone use, n (%)	429 (84)	148 (82)	259 (85)	21 (91)	0.325

Bold values indicate* p* < 0.05

*IQR* interquartile range, *SAPS2* simplified acute physiology score 2. *SOFA* sequential organ failure assessment. *VAP* ventilator-acquired pneumonia

In univariate analysis, patients from the “VAP with lung abscess” group received more probabilistic antibiotics at ICU admission and had a higher rate of confirmed bacterial coinfections.

Table 2 summarizes the main characteristics, microbiological details and treatments of patients that developed a lung abscess. Enterobacterales (15/23, 65%) were the main documentation followed by non-fermenting Gram-negative bacilli (10/23, 43%) and Gram-positive cocci (8/23, 35%). Lung abscesses were mainly plurimicrobial (15/23, 65%). Patients that secondarily developed a lung abscess had more frequently a plurimicrobial documentation during the 1st VAP episode as compared with patients that did not (11/23 (48%) vs. 86/303 (29%); p = 0.05).

**Table 2:** Clinical characteristics, radiological description, microbiological documentation and treatments in patients that developed a lung abscess

	VAP with lung abscess (n = 23)
Main delays, days, median (IQR)	
Time from beginning of MV to diagnosis of LA	18 (14–27)
Time from 1st VAP to diagnosis of LA	13 (4–23)
Number of VAP episodes	
One	8 (35)
Two	9 (39)
Three or more	6 (26)
Radiological data	
Reason for chest CT scan, n (%)	
LA suspicion	16 (70)
Other indication	7 (30)
Number of abscesses on chest CT scan, n (%)	
One	11 (48)
Two or more	12 (52)
Thoracic lateralization, n (%)	
Right lung	10 (43)
Left lung	6 (26)
Bilateral	7 (30)
Thoracic location	
Upper lobe	7 (30)
Lower lobe	8 (35)
Upper and lower lobes	8 (35)
Widest diameter, mm, median (IQR)	50 (37–80)
Visibility of LA on chest radiography before CT scan, n (%)	11 (48)
Associated pulmonary embolism or thrombosis, n (%)	8 (35)
History of pulmonary embolism or thrombosis during stay, n (%)	4 (17)
Microbiological data, n (%)	
Microbiological documentation (yes/no)	21 (91)
Number of bacteria involved	
One	8 (35)
Plurimicrobial	15 (65)
Bacterial documentation	
Enterobacterales	15 (65)
* Klebsiella spp.*	4 (17)
* Escherichia coli*	3 (13)
* Enterobacter spp.*	4 (17)
* Other*	1 (4)
Non-fermenting Gram-negative Bacilli	10 (43)
* Pseudomonas aeruginosa*	6 (26)
* Acinetobacter spp.*	3 (13)
* Stenotrophomonas maltophilia*	1 (4)
Gram-positive cocci	8 (35)
* Methicillin-susceptible Staphylococcus aureus*	6 (26)
* Methicillin-resistant Staphylococcus aureus*	1 (4)
* Enterococcus faecalis*	1 (4)
Anaerobes	
* Bacillus cereus*	1 (4)
Fungal documentation	9 (39)
* Aspergillus fumigatus*	8 (35)
Associated bacteremia	11 (48)
Antibiotic treatment	
Cephalosporins	17
Piperacillin–tazobactam	4
Carabapenems	8
Aminoglycosids	16
Ciprofloxacin	9
Vancomycin	2
Linezolid	6
Cotrimoxazole	5
Colistin	3
New beta-lactams (± inhibitors)^a^	6
Mono-therapy	0
Bi-therapy or more	23
Inhaled or instilled antibiotics	13
Antibiotic duration, days, median (IQR)	29 (15–43)
Thoracic drainage and/or decortication of pleural empyema	7 (30)
Complications, n (%)	
Pleural empyema	4 (17)
Pneumothorax	8 (35)
Control CT scan, n (%)	15 (65)
Time from LA diagnosis to 2nd CT scan (median, IQR)	14 (8–25)
Worsening	5 (33)
Unchanged	5 (33)
Improvement	5 (33)

*CT* computed tomography, *IQR* interquartile range, *LA* lung abscess, *MV* mechanical ventilation, *SAPS2* simplified acute physiology score 2, *SOFA* sequential organ failure assessment, *VAP* ventilator-acquired pneumonia

^a^Ceftolozane–tazobactam; ceftazidime–avibactam; cefiderocol

Four patients suffered from pleural empyema and 8 had a pneumothorax. None of them presented a hemoptysis. Seven patients needed a surgical treatment (thoracic drainage or pleural decortication).

### Risk factors for lung abscess

In multivariate analysis, 2 factors were independently associated with the development of a lung abscess: a plurimicrobial 1st VAP episode and the use of hydrocortisone (Table 3).

**Table 3:** Risk factors for lung abscess in multivariate analysis

	OR	95% CI	*p* value
Plurimicrobial 1st VAP episode	*2.925*	*1.164–7.511*	*0.022*
Probabilistic antibiotic therapy	2.662	0.938–7.554	0.066
Hydrocortisone during the ICU stay	*4.863*	*1.954–12.103*	*0.001*
Tocilizumab during the ICU stay	1.826	0.706–4.722	0.214

The model was adjusted with the effect of the center to reduce the selection bias.

### OR (odds ratio), CI (confidence interval).

#### Outcomes

Patients with lung abscesses had less VFD at day 60, a longer duration of MV, ICU and hospital stays than patients from the “VAP without lung abscess” group (respectively, 0 (0–3) vs. 16 (0–42) days; p < 0.001, 49 (32–73) vs. 25 (11–41) days; p < 0.001, 52 (36–77) vs. 28 (16–47) days; p < 0.001, 63 (42–96) vs. 36 (23–58) days; p < 0.001). Although higher than for no VAP group, mortality of patients with lung abscess was not significantly different from that in VAP without lung abscess group (12/23 (52%) vs. 104/303 (35%); p = 0.08) (Table 4).

**Table 4:** Patients’ outcomes

	All (n = 507)	No VAP (n = 181)	VAP without lung abscess (n = 303)	VAP with lung abscess (n = 23)	*P* value
ARDS severity					
PP, n (%)	398 (79)	122 (67)	255 (84)	21 (91)	< 0.001
Veno-venous ECMO, n (%)	96 (19)	23 (13)	61 (20)	12 (52)	< 0.001
Mortality, n (%)					
Day 28	85 (17)	31 (17)	52 (17)	2 (9)	0.50
Day 90	154 (30)	39 (22)	106 (41)*	9 (39)	0.008
ICU, n (%)	152 (30)	36 (20)	104 (35)*	12 (52)*	0.001
Hospital, n (%)	155 (31)	38 (21)	105 (35)*	12 (52)*	< 0.001
Mechanical ventilation duration, days					
VFD at Day 28 (median, IQR)	0 (0–17)	14 (0–21)	0 (0–11)*	0 (0–0)*	< 0.001
VFD at Day 60 (median, IQR)	27 (0–49)	46 (11–53)	16 (0–42)*	0 (0–3)*^#^	< 0.001
MV duration (median, IQR)	18 (9–36)	10 (6–19)	25 (11–41)*	49 (32–73)*^ #^	< 0.001
Length of stay, days					
ICU (median, IQR)	23 (13–42)	14 (9–25)	28 (16–47)*	52 (36–77)*^#^	< 0.001
Hospital (median, IQR)	31 (20–50)	24 (17–37)	36 (23–58)*	63 (42–96)*^#^	< 0.001

*ECMO* extracorporeal membrane oxygenation, *IQR* interquartile range, *MV* mechanical ventilation, *PP* prone positioning, *VAP* ventilated acquired pneumonia, *VFD* ventilator-free days, number of days living without mechanical ventilation

^*^p < 0.05 compared to the no VAP group

^#^p < 0.05 compared to VAP without lung abscess group

## Discussion

We describe herein a series of 23 patients among 326 (7%) that developed a lung abscess after VAP during COVID-19. This is one the first multicenter cohort focusing specifically on this complication. Plurimicrobial first VAP episode and treatment with hydrocortisone during the ICU stay were identified as factors associated with lung abscess occurrence.

In our cohort, the incidence of VAP was of 64% (326/507). Only 2 single-center studies specifically assessed lung abscesses incidence in COVID-19 patients with VAP. Beaucoté et al. [11] reported that 17 of 119 COVID-19 patients (14%) with VAP developed a lung abscess. More recently, Utsumi et al. [10] have found that 6/30 (20%) of patients with VAP developed pyothorax or lung abscess during COVID-19. In a systematic review [18] investigating VAP in ARDS following COVID-19, only one study [9] out of 16 reported the incidence of lung abscesses. In this work, the authors found a very low incidence (1.4%), but the study was not designed to document this complication. This variable incidence can be explained by the absence of systematic thoracic CT scan during ICU stay. Interestingly, in our series, a lung abscess was suspected on chest radiography in less than half cases, arguing for performing a CT scan in case of persisting VAP in COVID-19 patients.

Unsurprisingly, most lung abscesses were plurimicrobial. Enterobacterales were the most frequently retrieved bacteria, before *Pseudomonas aeruginosa* and *Staphylococcus aureus* which is in line with a previous report [11]. This bacteriological documentation is different to what is classically described in lung abscesses, in which anaerobes and *Streptococci* are frequent [19]. Of note, 8 patients (34%) had a probable COVID-19-associated pulmonary aspergillosis [14, 15]. Among them, all had at least one bacterial documentation of lung abscess. *Aspergillus fumigatus* was recovered from lung samples before or at the time of lung abscess diagnosis for 4 patients, suggesting a direct role in the development of lung abscess. In other cases, it is possible that *Aspergillus fumigatus* secondarily infected the pulmonary cavity.

Most patients (15/23) with lung abscesses had more than one episode of VAP. Recurrent VAP with mainly relapses despite adequate antibiotics have been described during COVID-19 [20]. One hypothesis is that the microvascular involvement during COVID-19 might alter the pulmonary diffusion of antibiotics resulting in persisting pneumonia, favoring later evolution toward lung abscess [5, 21].

Lung abscesses received prolonged antibiotics regimen, all of them with combinations of antibiotics. The use of inhaled or instilled treatments was frequent 13/23 (57%).

We aimed to identify factors associated with lung abscess development. Bacterial coinfection and probabilistic antibiotic treatment at ICU admission were more frequent in the lung abscess group. None of them appeared to be an independent risk factor. By contrast, a plurimicrobial documentation during 1st VAP episode was associated with further lung abscess occurrence. We also investigated the role of immunosuppressive or immunomodulatory treatments. Among them, only hydrocortisone was identified as an independent risk factor for lung abscesses. In non-COVID-19 indications, several studies have shown an increased risk of infection with tocilizumab as compared with other immunosuppressive treatments [22, 23]. In the studies evaluating tocilizumab in ICU patients with severe COVID-19, higher risk of infection was not described [24, 25] although these studies did not report late onset VAP. The role of hydrocortisone is more intriguing and an association with patients’ severity due to septic shock cannot be ruled out.

In our study, the presence of an abscess was associated with a 52% ICU mortality although the difference with VAP without abscess group was not significant. Mortality in patients with lung abscess in other series ranges from 50 to 65% [10, 11]. Patients with lung abscesses had lower VFD at day 60 and a longer ICU stay.

Our study has some limitations. First, we conducted a retrospective study, but this design allowed us to include patients from several waves of pandemic. Second, the diagnosis of lung abscesses being made on chest CT, it is possible that some patients with abscesses were not diagnosed, thus underestimating the incidence. On the other side, the frequent use of CT scans among COVID-19 patients for the diagnosis of VAP might explain the increased number of lung abscesses diagnosed. Finally, data are lacking in our cohort especially COVID-19 vaccination rates but also long terms outcomes, in particular respiratory condition and quality of life.

## Conclusions

In this multicenter cohort, we found that lung abscess is not uncommon and affects 7% of patients that develop a VAP under invasive MV for COVID-19. A plurimicrobial first VAP and the use of hydrocortisone were associated with this complication. Patients with lung abscess exhibited a high ICU mortality and a prolonged invasive MV and ICU stay. The frequency of this complication in COVID-19 patients altogether with its implication on treatment and prognosis argue for its research by performing a chest CT scan especially in persisting VAP.

### Supplementary Information

**Additional file 1:** Immunosuppressive or immunomodulatory treatments use during the ICU stay.

## Data Availability

The datasets used and/or analyzed during the current study are available from the corresponding author on reasonable request.

## Abbreviations

ARDS: Acute respiratory distress syndrome
BAL: Bronchoalveolar lavage
GNB: Gram-negative Bacilli
GPC: Gram-positive Cocci
COVID-19: Corona Virus Disease 2019
ECMO: Extracorporeal membrane oxygenation
CI: Confidence interval
CT: Computed tomography
IQR: Interquartile range
IL6: Interleukin 6
IL 1: Interleukin 1
BMI: Body mass index
JAK: Janus kinase
OR: Odds ratio
VAP: Ventilator-acquired pneumonia
PDS: Protected distal sample
MV: Mechanical ventilation
MSSA: Methicillin-susceptible *Staphylococcus aureus*
MRSA: Methicillin-resistant *Staphylococcus aureus*
SAPS 2: Simplified acute physiology score 2
SD: Standard deviation
SOFA: Sequential organ failure assessment
VFD: Ventilator-free days

## References

[1] ref WHO Coronavirus (COVID-19) Dashboard [Internet]. Disponible sur: https://covid19.who.int/data

[2] COVID-ICU Group on behalf of the REVA Network and the COVID-ICU Investigators. Clinical characteristics and day-90 outcomes of 4244 critically ill adults with COVID-19: a prospective cohort study. Intensive Care Med 2021;47(1):60–73. 10.1007/s00134-020-06294-x PMC7674575 33211135

[3] Rouzé A, Martin-Loeches I, Povoa P, Makris D, Artigas A, Bouchereau M (2021). Relationship between SARS-CoV-2 infection and the incidence of ventilator-associated lower respiratory tract infections: a European multicenter cohort study. Intensive Care Med.

[4] Luyt CE, Sahnoun T, Gautier M, Vidal P, Burrel S, Pineton de Chambrun M (2020). Ventilator-associated pneumonia in patients with SARS-CoV-2-associated acute respiratory distress syndrome requiring ECMO: a retrospective cohort study. Ann Intensive Care.

[5] Ackermann M, Verleden SE, Kuehnel M, Haverich A, Welte T, Laenger F (2020). Pulmonary vascular endothelialitis, thrombosis, and angiogenesis in Covid-19. N Engl J Med.

[6] Jeannet R, Daix T, Formento R, Feuillard J, François B (2020). Severe COVID-19 is associated with deep and sustained multifaceted cellular immunosuppression. Intensive Care Med.

[7] Maes M, Higginson E, Pereira-Dias J, Curran MD, Parmar S, Khokhar F (2021). Ventilator-associated pneumonia in critically ill patients with COVID-19. Crit Care.

[8] Umamoto K, Horiba M (2023). Lung abscess as a secondary infection of COVID-19: a case report and literature review. J Infect Chemother.

[9] Blonz G, Kouatchet A, Chudeau N, Pontis E, Lorber J, Lemeur A (2021). Epidemiology and microbiology of ventilator-associated pneumonia in COVID-19 patients: a multicenter retrospective study in 188 patients in an un-inundated French region. Crit Care.

[10] Utsumi S, Ohshimo S, Ishii J, Nishikimi M, Shime N (2023). Lung abscess and pyothorax in critically ill COVID-19 patients: a single-center retrospective study. Crit Care Explor.

[11] Beaucoté V, Plantefève G, Tirolien JA, Desaint P, Fraissé M, Contou D (2021). Lung abscess in critically ill coronavirus disease 2019 patients with ventilator-associated pneumonia: a French monocenter retrospective study. Crit Care Explor.

[12] Le Gall JR, Lemeshow S, Saulnier F (1993). A new Simplified Acute Physiology Score (SAPS II) based on a European/North American multicenter study. JAMA.

[13] Vincent JL, Moreno R, Takala J, Willatts S, de Mendonça A, Bruining H, et al. The SOFA (Sepsis-related Organ Failure Assessment) score to describe organ dysfunction/failure. On behalf of the Working Group on Sepsis-Related Problems of the European Society of Intensive Care Medicine. Intensive Care Med 1996;22(7):707‑10. 10.1007/BF01709751 8844239

[14] Donnelly JP, Chen SC, Kauffman CA, Steinbach WJ, Baddley JW, Verweij PE (2020). Revision and update of the consensus definitions of invasive fungal disease from the european organization for research and treatment of cancer and the mycoses study group education and research consortium. Clin Infectious Diseases..

[15] Koehler P, Bassetti M, Chakrabarti A, Chen SCA, Colombo AL, Hoenigl M (2021). Defining and managing COVID-19-associated pulmonary aspergillosis: the 2020 ECMM/ISHAM consensus criteria for research and clinical guidance. Lancet Infect Dis.

[16] Leone M, Bouadma L, Bouhemad B, Brissaud O, Dauger S, Gibot S (2018). Hospital-acquired pneumonia in ICU. Anaesth Crit Care Pain Med.

[17] Kalil AC, Metersky ML, Klompas M, Muscedere J, Sweeney DA, Palmer LB (2016). Management of adults with hospital-acquired and ventilator-associated pneumonia: 2016 clinical practice guidelines by the infectious diseases society of America and the American Thoracic Society. Clin Infect Dis.

[18] Fumagalli J, Panigada M, Klompas M, Berra L (2022). Ventilator-associated pneumonia among SARS-CoV-2 acute respiratory distress syndrome patients. Curr Opin Crit Care.

[19] Bartlett JG (2012). Anaerobic bacterial infection of the lung. Anaerobe.

[20] Gragueb-Chatti I, Hyvernat H, Leone M, Agard G, Peres N, Guervilly C (2022). Incidence, outcomes and risk factors of recurrent ventilator associated pneumonia in COVID-19 patients: a retrospective multicenter study. J Clin Med.

[21] Berkman SA, Tapson VF (2021). COVID-19 and its implications for thrombosis and anticoagulation. Semin Respir Crit Care Med.

[22] Lang VR, Englbrecht M, Rech J, Nüsslein H, Manger K, Schuch F (2012). Risk of infections in rheumatoid arthritis patients treated with tocilizumab. Rheumatology.

[23] Pawar A, Desai RJ, Solomon DH, Santiago Ortiz AJ, Gale S, Bao M (2019). Risk of serious infections in tocilizumab versus other biologic drugs in patients with rheumatoid arthritis: a multidatabase cohort study. Ann Rheum Dis.

[24] Gupta S, Wang W, Hayek SS, Chan L, Mathews KS, Melamed ML (2021). Association between early treatment with tocilizumab and mortality among critically ill patients with COVID-19. JAMA Intern Med.

[25] Rosas IO, Bräu N, Waters M, Go RC, Hunter BD, Bhagani S (2021). Tocilizumab in hospitalized patients with severe Covid-19 pneumonia. N Engl J Med.

