# Supplementary material for: Lung abscess following ventilator-associated pneumonia during COVID-19: a retrospective multicenter cohort study
Source: Crit Care. 2023 Oct 4;27:385. doi: 10.1186/s13054-023-04660-x (PMC10552315; doi:10.1186/s13054-023-04660-x)
Supplement: Supplementary file 1 — Additional file 1: Immunosuppressive or immunomodulatory treatments use during the ICU stay. [file 13054_2023_4660_MOESM1_ESM.docx]

|  | **All (n =507)** | **No VAP (n= 181)** | **VAP without lung abscess**  **(n= 303)** | **VAP with lung abscess (n=23)** | ***P value*** |
| --- | --- | --- | --- | --- | --- |
| **Dexamethasone (n%)** | 429 (84) | 148 (82) | 259 (85) | 21 (91) | 0.325 |
| **Immunosuppressive/Immunomodulatory treatments, n(%)** | 443 (87) | 145 (80) | 274 (91) * | 23 (100) ^#^ | **<0.001** |
| Methylprednisolone | 137 (27) | 29 (16) | 95 (31) * | 13 (57) * | **<0.001** |
| Ruxolitinib | 20 (4) | 4 (2) | 15 (5) | 1 (4) | 0.566 |
| Anakinra | 16 (3) | 4 (2) | 12 (4) | 0 (0) | 0.383 |
| Tocilizumab | 135 (26) | 21 (12) | 104 (34) * | 10 (45) * | **<0.001** |
| Hydrocortisone hemisuccinate, n(%) | 122 (24) | 27 (15) | 81 (27) ^# ᶲ^ | 14 (61) * | **<0.001** |
| Association of several immunosuppressive/ immunomodulatory treatments | 280 (55) | 65 (36) | 196 (65) * | 19 (83) * | **<0.001** |

**Table S1**

Immunosuppressive or immunomodulatory treatments use during the ICU stay.

*VAP: ventilator associated pneumonia*

** P<0.001 vs. No VAP by Kruskall-Wallis post-hoc test*

*^#^ P<0.05 vs. No VAP by Kruskall-Wallis post-hoc test*

^ᶲ^ *P<0.001 vs. VAP with lung abscess by Kruskall-Wallis post-hoc test*
